# Supplementary material for: Economic burden and catastrophic cost among people living with sickle cell disease, attending a tertiary health institution in south-east zone, Nigeria
Source: PLoS One. 2022 Aug 23;17(8):e0272491. doi: 10.1371/journal.pone.0272491 (PMC9398014; doi:10.1371/journal.pone.0272491)
Supplement: Attachment — Submitted filename: Response to reviewers PLOSONE.docx [file pone.0272491.s001.docx]

**TO:**	THE EDITOR-IN-CHIEF

PLOS ONE

**FROM:**	MRS AMARACHUKWU CHARITY

CORRESPONDING AUTHOR: DEPARTMENT OF HEALTH ADMINISTRATION AND MANAGEMENT, UNIVERSITY OF NIGERIA ENUGU CAMPUS, ENUGU NIGERIA.

**DATE:**	30 OCTOBER, 2021

**RE: Manuscript Number:**

**TITLE: Economic Burden and Catastrophic Cost among People Living With Sickle Cell Disease, Attending a Tertiary Health Institution in South-East Zone, Nigeria.**

The authors wish to thank the reviewers for their comments about this manuscript and for the constructive critique and recommendations. Kindly see below a tabulated response to all comments raised by both reviewers. All responses are effected in the manuscript.

| **Reviewer comment** | **Author response** | **Page** |
| --- | --- | --- |
| **Reviewer 1** | | |
| 1.**(Line 25 & 146)**  "To study a sample of 149 SCD"; "The final sample size was 156". Authors should clearly define the sample size. | Thank you for your significant contribution. The minimum sample size calculated was 140, but after adjustments for non-response, it became 156. However, 149 participants completed the questionnaire. We have revised the manuscript to capture detailed explanations | 2,7,8 |
| 2. **(Line 123)**   "About 480 SCD patients attended the children clinic and about 400 patients attended the adult clinic in the last one year". How should the term: "in the last one year" be understood? | Thank you for drawing our attention here. About 480 children and 400 adult patients with SCD attended the clinic within a year (September 2014 to October 2015) according to the clinical records. | 7 |
| 3. (**Line 206)**   "Total direct cost per patient per month was (US$289.41±316.18), with in-patient direct cost constituting the bulk (US$247.34±260.99)". Is it possible for costs to be negative? The cost written in this way (US$289.41±316.18) I understand as cost form: - 26.77 to 605.59. | We subjected the data set to normality test and discovered the data involved were not normally distributed hence we resorted to the statistic of median and corresponding interquartile range. | 11 |
| 4. (**Line 216)**  Table 2. What do IPD and OPD mean? | The authors thank the reviewer for drawing our attention. We have included the full meanings. | 12 |
| **Reviewer 2** |  |  |
| Unfortunately, assigning social status according to the possession of RTV/ household equipment is incomprehensible. This is subjective data. Treatment costs are perfectly presented. However, we are not able to estimate the scale of the problem because the earnings of the study group have not been presented. Rtv/ household equipment says absolutely nothing about wealth. We have no point of reference. It is difficult for me to compare the cost of treatment in US dollars to the wealth in household appliances, not the amount of earnings in currency. The result is presented that the treatment expenditure is high for patients, and we do not know the patients' income. There is no data on earnings, so we cannot estimate whether the study group was poor or wealthy, nor are we able to say whether treatment is expensive for them or not. Unfortunately. | Thank you for your significant contribution and concern. The authors considered the fact that methods of assessing household socio-economic position or status can be categorized into 2 major types: Monetary and Non- monetary. Monetary (Income or expenditure) measures are well understood and more popular. However, criticisms have been observed over using monetary measures to evaluate socio-economic status (SES) especially in low and middle income countries (LMIC) like ours.  These are the challenges of using household income or expenditure as a proxy for classifying SES in LMIC:   1. Households are often times reluctant to divulge income information and under- reporting of income 2. Difficulty in converting farm products and household gift into income.   3. Measurement errors which are inevitable due to income and expenditure being based on recall memory.  4. Poor quality of income and expenditure in LMIC.  5. Prices of goods, normal interest rates for semi- durable or durable goods are difficult to discern when constructing consumption aggregates.  Given these challenges, proxy indicators have been developed like the wealth index, whereby wealth indices use information about household materials to create index of household wealth.  Filmer and Pritchett (2001) constructed an asset index by using Principal Component Analysis (PCA).The application of PCA allows researchers to convert series of ownership variables into SES. Collection of asset data have been claimed to be more reliable than income since it uses simple questions or direct observation interview and should therefore suffer less from recall or social desirability bias.  These formed the basis of our using ownership of household equipment other than income as a proxy for wealth assessment. So, the ownership of household equipment was used to classify households into the poorest, the very poor, the poor and the least poor. Table 5 described how catastrophic the spending was on the different socio-economic status as previously grouped. | 7 |
| In addition, data from 6 years ago are presented. This is a very long period and at the moment the situation may (does not have to be) completely different. | We believe that the data is still valid even after 6 years because all conditions that cause catastrophic spending among people living with sickle cell disease still exists in Nigeria. Out of pocket spending continues to persist as the major mode of payment for healthcare in Nigeria. |  |
| **Table 1**.  Quantity and frequency should be split into two columns or the column should be described differently | We have revised accordingly. | 10 |
| Many spaces are missing, especially in the introduction | We have revised accordingly. | 3-5 |

I thank you in advance for your review of the revised manuscript and look forward to a positive response

Sincerely

Amarachukwu
