# Supplementary material for: Economic burden and catastrophic cost among people living with sickle cell disease, attending a tertiary health institution in south-east zone, Nigeria
Source: PLoS One. 2022 Aug 23;17(8):e0272491. doi: 10.1371/journal.pone.0272491 (PMC9398014; doi:10.1371/journal.pone.0272491)
Supplement: Attachment — Submitted filename: Rebuttal_Plosone.docx [file pone.0272491.s002.docx]

**TO:**	THE EDITOR-IN-CHIEF

PLOS ONE

**FROM:**	MRS AMARACHUKWU CHARITY

CORRESPONDING AUTHOR: DEPARTMENT OF HEALTH ADMINISTRATION AND MANAGEMENT, UNIVERSITY OF NIGERIA ENUGU CAMPUS, ENUGU NIGERIA.

**DATE:**	13 MAY, 2022

**RE: Manuscript Number:**

**TITLE: Economic Burden and Catastrophic Cost among People Living With Sickle Cell Disease, Attending a Tertiary Health Institution in South-East Zone, Nigeria.**

The authors wish to thank the reviewers for their comments about this manuscript and for the constructive critique and recommendations. Kindly see below a tabulated response to all comments raised by both reviewers. All responses are effected in the manuscript.

| **Reviewer comment** | **Author response** | **Page** |
| --- | --- | --- |
| **Reviewer 2** |  |  |
| I still believe that comparing monetary and non-monetary types is not a good idea.  Personally, I have no problems with financial liquidity and I would definitely be able to take care of my own and my family's treatment, but according to your classification, you would assign me between the poor and the least poor, which has nothing to do with reality. I am afraid it may be the same in your work. Maybe it was worth using national data, e.g. on the minimum wage in Nigeria. Anything so that a person from different socio-cultural realities could receive hard data and draw their own conclusions. At the moment, it looks like your important work may not be taken seriously because many people, like me, may assign themselves to one of your groups and treats all of your work at a distance.  However, I understand that the point of view may depend on the point of sitting, but think about who the recipient is and what you want to get the message from. | Thank you for your significant contribution and concern. The authors considered the fact that methods of assessing household socio-economic position or status can be categorized into 2 major types: Monetary and Non- monetary.  Monetary (Income or expenditure) measures are well understood and more popular and that is where national data and minimum wage come into play. However, criticisms have been observed over using monetary measures to evaluate socio-economic status (SES) especially in low and middle income countries (LMIC) like ours.  These are the challenges of using household income or expenditure(monetary) as a proxy for classifying SES in LMIC:   1. Households are often times reluctant to divulge income information and under- reporting of income 2. Difficulty in converting farm products and household gift into income. 3. Measurement errors which are inevitable due to income and expenditure being based on recall memory. 4. Poor quality of income and expenditure in LMIC. 5. Prices of goods, normal interest rates for semi- durable or durable goods are difficult to discern when constructing consumption aggregates.   Given these challenges, proxy indicators have been developed like the wealth index, whereby wealth indices use information about household materials to create index of household wealth.  Filmer and Pritchett (2001) constructed an asset index by using Principal Component Analysis (PCA).The application of PCA allows researchers to convert series of ownership variables into SES. Collection of asset data have been claimed to be more reliable than income since it uses simple questions or direct observation interview and should therefore suffer less from recall or social desirability bias.  These formed the basis of our using ownership of household equipment other than income as a proxy for wealth assessment. So, the ownership of household equipment was used to classify households into the poorest, the very poor, the poor and the least poor.  So, in our study we did not compare monetary and non monetary rather we used non monetary measure because of the shortcomings of monetary measure stated above.  The minimum wage of Nigeria as of the time of the study was #18,000.00 Eighteen thousand naira ($90.34). It was not reported because we didn’t use monetary measure. | 9, 10 |
| **Reviewer 3** |  |  |
| This is a difficult paper to understand, and some of the methods need more detailing. Also the presentation of the data needs to be clearer, and the discussion more focused  **Major points** I am not sure that the population being studied is representative of the SCD population, as they are attending a tertiary unit, appear to have good electricity supply and >85% have a TV, fridge, and fan, and half have a computer. Of the adults half appear to have post secondary school education  I assume most SCD patients are seen in primary care if uncomplicated, and then in secondary, with the more severe (and wealthiest) accessing what I assume is more expensive tertiary care.  Given the average number of days of admission and the high inpatient costs, and frequency of admission in this group, these appear to constitute a more severe phenotype. Generalising from this may not be justified, and certainly merits addressing in the discussion.  The means of determining catastrophic health costs from the proportion of non food expenditure could be more clearly described in the methods (line 198-199). I note the comment and response to the reviewer 2, though see this method as needing greater justification and referencing in the paper. I was surprised that given (line 80) 70% of Nigerians live on <$1 per day the population studied in all quartiles significantly exceeds this. | Thank you for your significant contributions. Sorry for the ambiguity in our explanation. We shall try to make it clearer.  The population studied is a true representation of the SCD population in Enugu, though in a tertiary institution. There are just two hospitals in Enugu state where SCD patients seek specialist care with specialist adult and paediatric haematologist. That is the state teaching hospital where we conducted the pilot study and the Federal teaching hospital where we conducted the study. They are both tertiary hospitals. The truth is that those that can afford this tertiary care are those in the category listed that have good house gadgets and post secondary education, those underprivileged patients who cannot afford the tertiary care are just at the mercy of the primary health care givers who at most times lack the skills to take care of these patients.  That is in the ideal situation where needed care are available at the primary health care (PHC) level. In Nigeria, the case is different because most PHC’s are dilapidated and adequate care not assessed. To make it worse, SCD care is not usually available because of lack of trained personnel in the care of the complex blood disorder. There is no haematology care even in the secondary care hospitals/state district hospitals. Haematology care in Enugu state here is only gotten in the federal/state teaching hospitals and few private hospitals.  Severity was assessed using number of times patient needed care in the past one month. Table 1 presented that more than half of the respondents assessed care just once a month. Less than half of them assessed care more than twice.  Discussed extensively as response to Reviewer 2 | 7  11  15 |
|  |  |  |
| **Minor points** I was not clear on the sample size calculation, and what this was addressing    Patients seem to be having mainly monthly follow up, and longer intervals between visits would be preferred for stable patients- as this would also have an impact on cost.  I assume there is a (local or national) protocol being used- could this be referenced if so. There is also a Nigerian National Guideline for the Control and Management of Sickle Cell Disease 2014, that could be cited if being used. This states: <In Nigeria, sickle cell disease is among the ten (10) priority non-communicable diseases (NCDs) and it contributes significantly to both child and adult morbidity and mortality. By virtue of its population, Nigeria stands out as the most sickle cell endemic country in Africa with an annual infant death of 100,000 representing 8% of infant mortality in the country. It is also estimated that about 24% Nigerian adults have sickle cell trait.>>  There are two references for the prevalence and mortality of SCD in Nigeria, and 2.3% for prevalence seems high. Though the reference given (9) is from a paper on psychological burden. Similarly, the mortality given in line 56 gives mortality as about 5% before age 5. This seems low, and comes from a paper (ref 10) on parental attitude to SCD. Given how few adults there were in the sample, this potentially could indicate high mortality in childhood. Could the authors provide additional references to support these figures?  It would be helpful to know what the costs for standard medications for sickle are in a basic package- and whether newer more expensive treatments were being offered, as the basic package for support is usually not so expensive. And possibly describe what these medicines were, so these OPD costs can be understood (as medicines $15 USD/month). And why physiotherapy was over $20/ month median in OPD.    There are newer treatments for SCD such as hydroxyurea, and in the context of the costs for these patients would be worth considering as a possible means to reduce admissions and may be cost effective in reducing costs associated with more severe disease. The authors may want to provide estimate for the cost of these treatments for an adult per month- though this is above the remit of the paper, it does illustrate the decisions clinicians should have with their patients who have such large OOOP expenditures. Though these in themselves may also put heavy burden on families. | Thank you for your significant contribution. The minimum sample size calculated was 140, but after adjustments for non-response, it became 156. However, 149 participants completed the questionnaire. We have revised the manuscript to capture detailed explanations.  Follow up is mainly on patients need not on cost implication. Table 1 presented that more than half of the respondents assessed care just once a month. Less than half of them assessed care more than twice.  The authors thank the reviewer for drawing our attention. We have included the national guideline for the control and management of Sickle cell Disease in Nigeria, 2014 as an extra reference.  We have included another study in support of the 2.3% prevalence.  Studies seen that documented 50-90% under-five mortality were either after 2016 or across Africa not Nigeria. One study done in 2013 covering Africa was added.  The basic package comprises of:  Folic acid, Pain medication, Antibiotics, Malaria prophylaxis and/or treatment, Multivitamins, Omega 3 fatty acid, Occasionally Normal saline.  The above costs about $15.06-25.10USD/month per person. It’s good to state that OPD cost is more than just medicine but comprises cost of other items as listed in table. This cost of physiotherapy was said to be average. However, the fact that that physiotherapy cost higher than OPD medicine may reflect a culture of poor health seeking behaviour in Nigeria in which patients fail to take continue preventive/treatment measures until illness ensue or exacerbated. Nevertheless, the fact that only a few participants received physiotherapy may reflect the high cost, as there is tendency for exaggeration when sample size is small.  Hydroxyurea was not routinely prescribed to the patients as of the time of the study. The Haematologists opined that it is used based on severity of the condition because of its side effects and costs. | 8  11,12  3  3 |
|  |  |  |
| **There are a few typos or potential corrections** |  |  |
| Lines 174 and 175 – is the low reliability coefficient of 0.23 in group A or D. | Now line 183-184 revised accordingly |  |
| Line 207. I assume ….had SCD diagnosed for one to ten years. | Now line 217 revised accordingly |  |
| Table 1. Patient age box has 148 patients, as does number of times patients needed care (though n=149). And Variable 4 I think is ‘Age at SCD diagnosis’ rather than ‘Time since diagnosis’ | Part 2 revised accordingly |  |
| Line 216-218 are repeated. And (line 217) if 28 of the 149 respondents were admitted in the month studied, I struggled to understand how the median costs was $385, as most patients would have only incurred OPD costs- it would be helpful to understand how this small proportion of admissions raised the median cost so much. | The authors thank the reviewer for his/her input. Despite the fact that fewer patients were admitted, as expected, inpatient cost usually far outweighs outpatient cost. As can be seen from Table 2, the median inpatient cost was 338.6 while the median outpatient cost was 72.14. This signifies that admission raised the total cost by over 80%, and this is justified by the high admission cost. |  |
| Line 253 US$33 should be US$332 (from table 5) | Revised accordingly |  |

I thank you in advance for your review of the revised manuscript and look forward to a positive response

Sincerely

Amarachukwu
